# Integrated pest management with stochastic birth rate for prey species

**DOI:** 10.3389/fnins.2013.00141

**Published:** 2013-08-08

**Authors:** Olcay Akman, Timothy D. Comar, Daniel Hrozencik

**Affiliations:** ^1^Department of Mathematics, Illinois State UniversityNormal, IL, USA; ^2^Department of Mathematics, Benedictine UniversityLisle, IL, USA; ^3^Department of Mathematics and Computer Sciences, Chicago State UniversityChicago, IL, USA

**Keywords:** predator-prey interactions, eradication, permanent suppression, stability, environment

## Abstract

Song and Xiang (2006) developed an impulsive differential equations model for a two-prey one-predator model with stage structure for the predator. They demonstrate the conditions on the impulsive period for which a globally asymptotically stable pest-eradication periodic solution exists, as well as conditions on the impulsive period for which the prey species is permanently maintained under an economically acceptable threshold. We extend their model by including stage structure for both predator and prey as well as by adding stochastic elements in the birth rate of the prey. As in Song and Xiang (2006), we find the conditions under which a globally asymptotically stable pest eradication periodic solution exists. In addition, we numerically show the relationship between the stochastically varying birth rate of the prey and the necessary efficacy of the pesticide for which the probability of eradication of the prey species is above 90%. This is significant because the model recognizes varying environmental and climatic conditions which affect the resources needed for pest eradication.

## 1. Introduction

It is well-known that a variety of pest species pose a serious health risk to humans and pets, as well as causing great damage to property and crops. For virtually all pest species, biological eradication is biologically impossible or economically infeasible (Zhang et al., 2007). However, it has been shown that with an integrated pest management (IPM) approach, utilizing combinations of pesticides, predator species, and prey disease, prey species can be controlled at economically and environmentally feasible levels. The IPM approach has been proven superior to either purely biological control or chemical control (Song and Xiang, 2006).

A number of recent articles have mathematically modeled a variety of IPM approaches using impulsive differential equations, taking into account, for example, stage structure in the predator species and periodically varying environmental conditions (Song and Xiang, 2006). In the current literature, similar models also have been considered (Tang et al., 2005; Zhang et al., 2007). These deterministic models assume fixed birth rates for the prey species. As is more realistic in most ecosystems, we consider a random birth rate following a prior distribution with a mean that replaces the fixed birth rate of the previous models considered in Zhang et al. (2003, 2005, 2007); Tang et al. (2005); Song and Xiang (2006). This approach generalizes the model to accommodate random fluctuations, not just periodic fluctuations, in the birth rate due to environmental and climatic factors. In ecosystems, it is common for the reproductive behavior and fecundity of insect species to be altered by varying environmental and climatic factors such as temperature, light levels, and day length (Paulson et al., 2009). The stochastic birth rate component in the proposed model accommodates factors such as shortened day length and lower temperatures, which may induce varying levels of egg production (Paulson et al., 2009). Also, it recognizes that a fixed birth rate really represents an “average” birth rate, which may produce misleading results as to the resources necessary to ensure a high probability of pest eradication. This is most important in cases in which the population of the prey species is especially sensitive to changes in the potency of the pesticide. This semi-deterministic method is a novel approach. It can be applied to cases in which *a priori* information is available for birth rate distributions, even if it is not informative. The results we obtain will provide information on the values of other parameters that will ensure a high probability of eradication of the prey species under varying birth rates of the prey species.

The present paper is organized as follows: In Section 2, we discuss our impulsive differential equations model, introducing the essential variables and parameters. In Section 3, we use Floquet theory and results from Song and Xiang (2006) to establish conditions on the impulsive period for which our pest eradication solution is (i) locally asymptotically stable and (ii) globally asymptotically stable. In Section 4, we introduce a right-skewed distribution for the birth rate parameter b for the prey species. We present numerical results showing the relationship between the birth rate parameter b and value of E, the pesticide potency or application effectiveness.

## 2. The deterministic model

Our deterministic model consists of a prey species with a juvenile class *x*_1_ and a mature class *x*_2_, and a predator species with a juvenile class *y*_1_ and a mature class *y*_2_. The prey species is born periodically at time intervals of length *T* via a Ricker-type birth pulse *b* exp(−(*x*_1_(*nT*) + *x*_2_(*nT*))*x*_2_(*nT*), where *b* is the growth parameter, as considered in the model in Tang and Chen (2002). Immediately after the births, pesticide is sprayed, which kills a fraction *E* of both the juvenile and mature prey classes, whereas the two predator classes *y*_1_ and *y*_2_ are augmented by *p*_1_ and *p*_2_, respectively. The prey population is also decreased due to predation by the mature predators only, with parameter *r* > 0. The handling time of both *x*_1_ and *x*_2_ by the predator is *h*, and the conversion rate of killed prey in excess of what is needed for maintenance into births of new predators is λ. For instance, if λ = 0, then there is no effect of kills on predator births. Similarly, for very small positive values of λ, the efficiency of conversion is minimal. This conversion rate expression was also used in the models in Tang et al. (2005); Song and Xiang (2006). The maturity rates for the prey and predator species are *m*_*x*_ and *m*_*y*_, respectively. That is, 1/*m*_*i*_ is the mean length of the juvenile period. The death rate of the predator is μ. The model equations are given by

$$\begin{array}{l} \begin{array}{l} x_{1}^{\prime }(t)=-m_{x}x_{1}(t)-rx_{1}y_{2}(t)\\ x_{2}^{\prime }(t)=m_{x}x_{1}(t)-rx_{2}y_{2}(t)\\ y_{1}^{\prime }(t)=\frac{\lambda r(x_{1}(t)+x_{2}(t))y_{2}(t)}{1+rh(x_{1}(t)+x_{2}(t))}-(m_{y}+\mu )y_{1}(t)\\ y_{2}^{\prime }(t)=m_{y}y_{1}(t)-\mu y_{2}(t) \end{array} \end{array}\}\;t\neq nT,$$

(1) $$\begin{array}{l} \begin{array}{l} x_{1}(t^{+})=(x_{1}(t)+b\exp(-(x_{1}(t)\\ \;+x_{2}(t))x_{2}(t)))(1-E)\\ x_{2}(t^{+})=x_{2}(t)(1-E)\\ y_{1}(t^{+})=y_{1}(t)+p_{1}\\ y_{2}(t^{+})=y_{2}(t)+p_{2} \end{array} \end{array}\}\;t=nT.$$

This is a system of impulsive differential equations, which we consider only in the biologically meaningful domain *D* = {(*x*_1_, *x*_2_, *y*_1_, *y*_2_) | *x*_1_ ≥ 0, *x*_2_ ≥ 0, *y*_1_ ≥ 0, *y*_2_ ≥ 0}. For details on the theory of impulsive differential equations, we refer to the reader to the monograph (Lakshmikantham et al., 1989). For periodic solutions of such impulsive differential equations, see Bainov and Simenov (1993). Furthermore, Lemmas 3.1 and 3.2 provide simple examples of such periodic solutions.

## 3. Stability

We will need the following lemmas for the arguments in this section.

**Lemma 3.1** (Song and Xiang, 2006). *The system*

(2) $$\begin{array}{l} \;u^{\prime }(t)=a-bu(t),\;t\neq nT\\ u_{1}(t^{+})=u_{1}(t)+p,\;t=nT\\ u_{1}(0^{+})=u_{0}\geq 0 \end{array}$$

*has a unique positive, periodic, globally asymptotic solution ũ with period *T*, given by*

$$\tilde{u}=\frac{a}{b}+\frac{p\exp(-b(t-nT))}{1-\exp(-bT)},\;nT<t\leq (n+1)T,n\in N,$$

*and*

$$\tilde{u}(0^{+})=\frac{a}{b}+\frac{p}{1-\exp(-bT)}.$$

*For any other solution u*(*t*) *of the system, we have* |*u*(*t*) − ũ(*t*)| → 0 *as t* → ∞.

**Lemma 3.2** (Song and Xiang, 2006). *Consider the subsystem*

$$\begin{array}{l} \begin{array}{l} y_{1}^{\prime }(t)=-(m_{y}+\mu )y_{1}(t)\\ y_{2}^{\prime }(t)=m_{y}y_{1}(t)-\mu y_{2}(t) \end{array} \end{array}\}\;t\neq nT$$

(3) $$\begin{array}{l} \begin{array}{l} y_{1}(t^{+})=y_{1}(t)+p_{1}\\ y_{2}(t^{+})=y_{2}(t)+p_{2} \end{array} \end{array}\}\;t=nT.$$

*The subsystem (3) has the positive, periodic, globally asymptotic solution*

$$\begin{array}{l} {\tilde{y}}_{1}(t)=\frac{p_{1}\exp(-(m_{y}+\mu )(t-nT))}{1-\exp(-(m_{y}+\mu )T)}\\ {\tilde{y}}_{2}(t)=\frac{\left(p_{1}+p_{2})\exp(-\mu (t-nT)\right)}{1-\exp(-\mu T)}\\ \;-\frac{p_{1}\exp(-(m_{y}+\mu )(t-nT))}{1-\exp(-(m_{y}+\mu )T)} \end{array}\}nT<t\leq (n+1)T,$$

*with initial values*

(4) $$\begin{array}{l} {\tilde{y}}_{1}(0^{+})=\frac{p_{1}}{1-\exp(-(m_{y}+\mu )T)}\\ {\tilde{y}}_{2}(0^{+})=\frac{p_{1}+p_{2}}{1-\exp(-\mu T)}-\frac{p_{1}}{1-\exp(-(m_{y}+\mu )T)}. \end{array}\}$$

**Theorem 3.1**. *The pest eradication periodic solution* (0, 0, ỹ_1_(*t*), ỹ_2_(*t*)) *of system (1) is locally asymptotically stable if*

$$T<\frac{1}{m_{x}}\ln\left(\frac{(b-1)(1-E)+\exp(-rN){\left(1-E\right)}^{2}}{\left(1-E)(1+b)-\exp(rN\right)}\right),$$

*or equivalently, if*

$$b<\frac{\left(1-\exp(-rN)(1-E))(1-\exp(-m_{x}T-rN)(1-E)\right)}{\exp(-rN)(1-\exp(-m_{x}T))(1-E)},$$

*and globally asymptotically stable if*

$$b<\frac{1-\exp(-rN)(1-E)}{\exp(-rN)(1-E)},$$

*where*

$$N=\frac{\mu p_{2}+m_{y}(p_{1}+p_{2})}{\mu (m_{y}+\mu )}.$$

**Proof**. We first prove that the solution is locally asymptotically stable using Floquet Theory for impulsive differential equations (see Bainov and Simenov, 1993). We begin by taking a small amplitude perturbation (*u*_1_(*t*), *u*_2_(*t*), *ỹ*_1_(*t*) + *v*_1_(*t*), *ỹ*_2_(*t*) + *v*_2_(*t*)) of the pest eradication solution (0, 0, *ỹ*_1_(*t*), *ỹ*_2_(*t*)). Linearizing, we obtain the system

$$\frac{d\Phi (t)}{dt}=\left[\begin{array}{cccc} -m_{x}-r{\tilde{y}}_{2}(t) & 0 & 0 & 0\\ m_{x} & -r{\tilde{y}}_{2}(t) & 0 & 0\\ \lambda r{\tilde{y}}_{2}(t) & \lambda r{\tilde{y}}_{2}(t) & -(m_{y}+\mu ) & 0\\ 0 & 0 & m_{y} & -\mu \end{array}\right]\Phi (t),$$

where Φ(*t*) is the fundamental solution matrix of the system with Φ(0) = *I*, the identity matrix. The linearization of the pulse behavior is given by

$$P=\left[\begin{array}{cccc} 1-E & b(1-E) & 0 & 0\\ 0 & 1-E & 0 & 0\\ 0 & 0 & 1 & 0\\ 0 & 0 & 0 & 1 \end{array}\right].$$

Hence, the monodromy matrix of the system is

(5) $$\begin{array}{l} M=P\Phi (T)\\ \;=\left[\begin{array}{cccc} \left(\phi_{1}+b(\phi_{2}-\phi_{1}))(1-E\right) & b\phi_{2}(1-E) & 0 & 0\\ \left(\phi_{2}-\phi_{1})(1-E\right) & \phi_{2}(1-E) & 0 & 0\\ {}** & * & \exp(-(m_{y}+\mu )T) & 0\\ {}** & * & * & \exp(-\mu T) \end{array}\right], \end{array}$$

where

$$\phi_{1}=\exp\left(-\int_{0}^{T}\left(m_{x}+r{\tilde{y}}_{2}\left(t\right)\right)\right)dt$$

and

$$\phi_{2}=\exp\left(-\int_{0}^{T}r{\tilde{y}}_{2}(t)\right)dt.$$

By Floquet Theory, the solution is locally asymptotically stable if the absolute values of the eigenvalues of *M* are less than one. This is always the case for the two eigenvalues exp(−(*m*_*y*_ + μ)*T*) and exp(−μ *T*). The other two eigenvalues are the eigenvalues of the submatrix

(6) $$\bar{M}=\left[\begin{array}{cc} \left(\phi_{1}+b(\phi_{2}-\phi_{1}))(1-E\right) & b\phi_{2}(1-E)\\ \left(\phi_{2}-\phi_{1})(1-E\right) & \phi_{2}(1-E) \end{array}\right].$$

The two eigenvalues of the matrix $\bar{M}$ are less than one in absolute value if (see Li and Yang, 2011)

(7) $$\det(\bar{M})=\phi_{1}\phi_{2}{\left(1-E\right)}^{2}<1$$

(8) $$\text{trace}(\bar{M})-1-\det(\bar{M})=(\phi_{1}+\phi_{2}+b(\phi_{2}-\phi_{1}))(1-E)-1-\phi_{1}\phi_{2}{\left(1-E\right)}^{2}<0.$$

Clearly, inequality (7) is always satisfied, and inequality (8) is satisfied by the hypothesis. Hence, the solution is locally asymptotically stable.

We next prove the global attractivity of our solution following the technique in Song and Xiang (2006). Choose ϵ_1_ > 0 and ϵ_2_ > 0 sufficiently small so that

$$\delta =(1-E)(1+b)\;\exp\left(r\epsilon_{2}T-r\left(\frac{p_{1}+p_{2}}{\mu }-\frac{p_{1}}{m_{y}+\mu }-\frac{m_{y}\epsilon_{1}T}{\mu }\right)\right)<1.$$

We observe that

$${{\tilde{y}}^{\prime }}_{1}(t)\geq -(m_{y}+\mu )y_{1}(t),$$

and consider the following comparison impulsive differential equation:

(9) $$\begin{array}{l} \;{z^{\prime }}_{1}(t)=-(m_{y}+\mu )z_{1}(t),\;t\neq nT\\ \;z_{1}(t^{+})=z_{1}(t)+p_{1},\;t=nT\\ \;z_{1}(0^{+})=y_{1}(0^{+})\geq 0. \end{array}$$

By Lemma 3.1, system (9) has a globally asymptotically stable, positive, periodic solution

$${\tilde{z}}_{1}(t)=\frac{p_{1}\exp(-(m_{y}+\mu )(t-nT))}{1-\exp(-(m_{y}+\mu )T)},\;nT\;<\;t\;\leq \;(n+1)T.$$

By the Comparison Lemma for impulsive differential equations (see Lakshmikantham et al., 1989), we have

(10) $$y_{1}(t)\;\geq \;z_{1}(t)\;>\;{\tilde{z}}_{1}(t)-\epsilon_{1}.$$

From this inequality, we obtain

$${y^{\prime }}_{2}(t)\;\geq \;m_{y}({\tilde{z}}_{1}(t)-\epsilon )-\mu y_{2}(t).$$

We next consider the comparison system

(11) $$\begin{array}{l} \;{z^{\prime }}_{2}(t)=-m_{y}({\tilde{z}}_{1}(t)-\epsilon )-\mu z_{2}(t),\;t\neq nT\\ \;z_{2}(t^{+})=z_{2}(t)+p_{2},\;t=nT\\ \;z_{2}(0^{+})=y_{2}(0^{+})\;\geq \;0. \end{array}$$

By direct calculation, we observe that for *nT* < *t* ≤ (*n* + 1)*T,*

$${\tilde{z}}_{2}(t)=-\frac{p_{1}\exp(-(m_{y}+\mu )(t-nT))}{1-\exp(-(m_{y}+\mu )T)}+\frac{\left(p_{1}+p_{2})\exp(-(\mu (t-nT))\right)}{1-\exp(-\mu T)}-\frac{m_{y}\epsilon_{1}}{\mu }$$

is a positive, periodic, globally asymptotically stable solution of system (11). Again by the Comparison Lemma, we have

(12) $$y_{2}(t)\;\geq \;z_{2}(t)\;>\;{\tilde{z}}_{2}(t)-\epsilon_{2}$$

for sufficiently large *t*.

Now let *w*(*t*) = *x*_1_(*t*) + *x*_2_(*t*). From the first two equations of system (1), we obtain

(13) $$w^{\prime }(t)\;\leq \;-rw(t)({\tilde{z}}_{2}(t)-\epsilon_{2})$$

for *nT* < *t* ≤ (*n* + 1)*T*, and

(14) $$w(t^{+})\;\leq \;w(t)(1+b\exp(-w(t)))(1-E)$$

(15) ≤w(t)(1+b)(1−E)

for *t* = *nT*. For *w*(*t*), we consider the comparison system

(16) $$\begin{array}{l} \;{z^{\prime }}_{3}(t)=-rz_{3}(t)({\tilde{z}}_{2}(t)-\epsilon_{2}),\;t\neq nT\\ \;z_{3}(t^{+})=z_{3}(t)(1+b)(1-E),\;t=nT\\ \;z_{3}(0^{+})=w(0^{+})=x_{1}(0^{+})+x_{2}(0^{+})\geq 0. \end{array}$$

By integrating from *t* = *nT*^+^ to *t* = (*n* + 1)*T*, we obtain

(17) $$z_{3}((n+1)T)=z_{3}(nT^{+})\exp\left(r\epsilon_{2}T-r\int_{nT}^{(n+1)T}{\tilde{z}}_{2}(t)dt\right),$$

where

$$\int_{nT}^{(n+1)T}{\tilde{z}}_{2}(t)dt=\frac{\left(p_{1}+p_{2}\right)}{\mu }-\frac{p_{1}}{m_{y}+\mu }-\frac{m\epsilon_{1}T}{\mu }.$$

We now obtain the stroboscopic map

(18) $$\begin{array}{l} z_{3}((n+1)T^{+})=z_{3}(nT^{+})(1+b)(1-E)\exp\left(r\epsilon_{2}T-r\int_{nT}^{(n+1)T}{\tilde{z}}_{2}(t)dt\right)\\ \;=z_{3}(nT^{+})\delta . \end{array}$$

Hence, *z*_3_(*nT*^+^) = δ^*n*^*z*_3_(0^+^), and *z*_3_(*nT*^+^) → 0 as *n* → ∞. Equation (18) has the unique equilibrium z^*^_3_ = 0, which is globally asymptotically stable. Thus, system (16) has the globally asymptotically stable solution ${\tilde{z}}_{3}(t)=0$. We can conclude that lim_*t* → ∞_ *w*(*t*) = 0, and hence lim_*t* → ∞_ *x*_1_(*t*) = 0 and lim_*t* → ∞_*x*_2_(*t*) = 0, since *x*_1_(*t*) ≥ 0 and *x*_2_(*t*) ≥ 0.

We next show that lim_*t* → ∞_ *y*_1_(*t*) = 0 and lim_*t* → ∞_ *y*_2_(*t*) = 0. For sufficiently small ϵ_3_ > 0, there exists *T*_1_ > 0 such that

0 < *x*_1_(*t*) < ϵ_3_ and 0 < *x*_2_(*t*) < ϵ_3_ for all *t* > *T*_1_. The function

$$\frac{\lambda r\;w(t)}{1+rh\;w(t)}$$

is monotonically increasing for *w*(*t*) ≥ 0. Let

$$K=\frac{\lambda r\epsilon_{3}M}{1+rh\epsilon_{3}}.$$

We now have

$$y_{1}(t)\leq K-(m_{y}+\mu )y_{1}(t).$$

Consider the comparison system

(19) $$\begin{array}{l} \;{z^{\prime }}_{4}(t)=K-(m_{y}+\mu )z_{4}(t),\;t\neq nT\\ \;z_{4}(t^{+})=z_{4}(t)+p_{1},\;t=nT\\ \;z_{4}(0^{+})=y_{1}(0^{+})\;\geq \;0. \end{array}$$

By Lemma 3.1, this comparison system has the positive, periodic, globally asymptotically stable solution

$${\tilde{z}}_{4}(t)=\frac{p_{1}\exp(-(m_{y}+\mu )(t-nT))}{1-\exp(-(m_{y}+\mu )T}+\frac{K}{m_{y}+\mu }.$$

Hence, for sufficiently small ϵ_4_ > 0 and large enough *t*, we have

(20) $$y_{1}(t)\;\leq \;z_{4}(t)\;<\;{\tilde{z}}_{4}(t)+\epsilon_{4}.$$

From the inequalities (10) and (20), we obtain

$${\tilde{z}}_{1}(t)-\epsilon_{1}\;<\;y_{1}(t)\;<\;{\tilde{z}}_{4}(t)+\epsilon_{4}$$

for sufficiently large *t*. Letting ϵ_1_ → 0, ϵ_3_ → 0, and ϵ_4_ → 0, we obtain ${\tilde{z}}_{1}(t)\to {\tilde{y}}_{1}(t)$ and ${\tilde{z}}_{4}(t)\to {\tilde{y}}_{1}(t)$ as *t* → ∞. Hence, lim_*t* → ∞_ *y*_1_(*t*) = *ỹ*_1_(*t*).

Using the fourth equation from system (1) and the inequality (20), we obtain the inequality

$${y^{\prime }}_{2}(t)\;\leq \;m_{y}({\tilde{z}}_{4}(t)+\epsilon_{4})-\mu y_{2}(t).$$

For this inequality, we consider the comparison system

(21) $$\begin{array}{l} \;{z^{\prime }}_{5}(t)=m_{y}({\tilde{z}}_{4}(t)+\epsilon_{4})-\mu z_{5}(t),\;t\neq nT\\ \;z_{5}(t^{+})=z_{5}(t)+p_{2},\;t=nT\\ \;z_{5}(0^{+})=y_{2}(0^{+})\;\geq \;0. \end{array}$$

This system has a periodic, globally asymptotically stable solution

$${\tilde{z}}_{5}(t)=-\frac{p_{1}\exp(-(m_{y}+\mu )(t-nT)}{1-\exp(-(-m_{y}+\mu )T)}+\frac{\left(p_{1}+p_{2})\exp(-\mu (t-nT)\right)}{1-\exp(-\mu T)}+\frac{m_{y}}{\mu }\left(\frac{K}{m_{y}+\mu }+\epsilon_{4}\right)$$

for *nT* < *t* ≤ (*n* + 1)*T*. By the Comparison Lemma, we have

(22) $$y_{2}(t)\;\leq \;z_{5}(t)\;<\;{\tilde{z}}_{5}(t)+\epsilon_{5}$$

for sufficiently large *t*. The inequalities (12) and (22) imply that

$${\tilde{z}}_{2}(t)-\epsilon_{2}\;<\;y_{2}(t)\;<\;{\tilde{z}}_{5}(t)+\epsilon_{5}$$

for sufficiently large *t*. Letting ϵ_2_ → 0, ϵ_3_ → 0, and ϵ_5_ → 0, we obtain ${\tilde{z}}_{2}(t)\to {\tilde{y}}_{2}(t)$ and ${\tilde{z}}_{5}(t)\to {\tilde{y}}_{2}(t)$ as *t* → ∞. Hence, lim_*t* → ∞_ *y*_2_(*t*) = ỹ_2_(*t*).        □

## 4. Discussion

### 4.1. Stochastic birth rate model

In this paper, we consider an integrated pest management (IPM) model with two stages for both predator and prey, where prey births occur according to a birth pulse. We found conditions for global stability of the pest eradication periodic solution. In particular, we express this relationship in terms of an upper bound on b, the parameter in the birth pulse expression.

We now turn to the stochastic model. Specifically, we consider the birth rate parameter *b* of the prey species given in system (1) to be random. As special case, we consider *b* to follow a right-skewed distribution reflecting the ecosystem where small birth rates are prevalent, while due to climatic changes, infrequent but large spikes in the birth rate are probable. However, the approach employed here can be easily implemented if *a priori* information indicates a different birth rate behavior. In fact, even if no information regarding the nature of the birth rate of the prey species is available, one can still implement the model given herein by simply using a uniform distribution for the birth rate, where all possible values of the birth rate are equally likely.

The model in Song and Xiang (2006) considers only periodically varying environments, where the environmental and climatic conditions follow a predictable pattern. Our model includes the behavior of this model as a particular instance. In other words, we extend the model given in Song and Xiang (2006) such that environmental and climatic conditions of a more random nature can be modeled.

## 5. Conclusions and remarks

Here we seek a value of *E*, the pesticide potency or application effectiveness, under randomly varying birth rates in an attempt to maximize the eradication probability. Our approach with stochastic birth rate parameter forms a class of models, which accommodate a wide spectrum of cases where the eradication probability depends not only on the pesticide use but also on the abundance of the prey species.

**Figure 1:**
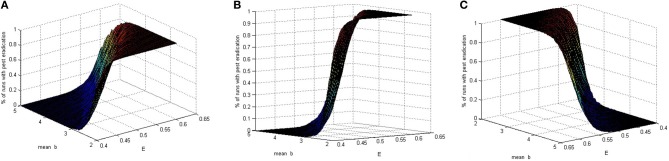
**Three views of the percentage of runs with eradication for ordered pairs (*E, b*), where *b* is stochastic and *E* is deterministic**.

In a model with deterministic birth rate, whether the eradication occurs depends on the model parameters. Using our model, with the introduction of a stochastic parameter, we capture the process of varying birth rates that result in a realization for *b*, while holding all other values fixed and then compute the probability of eradication. To be more specific, we simulate a population of exponentially distributed birth rate parameters with mean *b*. Next we check the proportion of runs for which the total pest population is below a predetermined tolerance after a given length of time, which we define as eradication. Figure 1 depicts the probability of eradication for given values of *E* and mean of *b* values. In this particular case when the magnitude of the prey birth rate follows a right-skewed random behavior with varying averages, the pesticide amount or potency may be determined based on the eradication probability. As expected, large pesticide values while the birth rates vary around a small mean result in high eradication probability.

The graph in Figure 1 is obtained using arbitrarily chosen fixed parameter values *m*_*x*_ = 0.8, *m*_*y*_ = 0.7, *r* = 0.1, μ = 0.2, *h* = 0.5, λ = 0.1, *p*_1_ = 0.4, *p*_2_ = 0.3, and *T* = 1. With these values and *E* = 0.5, local asymptotic stability of the pest eradication solution occurs when *b* < 2.5985428. This is consistent with the results shown in Figure 1.

We began this study by considering an integrated pest management IPM model in which prey births occur according to a deterministic birth pulse. We established conditions for global stability of the pest eradication solution in terms of the birth rate parameter *b* of the prey species. We modified the model by considering *b* to be random. The stochastic version of our model extends the model in Song and Xiang (2006) by allowing for random environmental and climatic conditions. The stochastic model more clearly explains the relationship between the birth rate parameter *b* and the efficacy of the pesticide *E* needed for pest eradication.

### Conflict of interest statement

The authors declare that the research was conducted in the absence of any commercial or financial relationships that could be construed as a potential conflict of interest.
